# Genetic characterization and genome-wide association mapping for stem rust resistance in spring bread wheat

**DOI:** 10.1186/s12863-022-01030-4

**Published:** 2022-02-14

**Authors:** Elias Shewabez, Endashaw Bekele, Admas Alemu, Laura Mugnai, Wuletaw Tadesse

**Affiliations:** 1 Department of Microbial, Cellular and Molecular Biology, Addis Ababa University, P.O. Box 1176, Addis Ababa, Ethiopia; 2 Department of Biology, Debre Tabor University, P.O. Box 272, Debre Tabor, Ethiopia; 3 Department of Agriculture, Food, Environment and Forestry, University of Florence, Piazzale delle Cascine 18 - 50144, Firenze, FI Italy; 4 International Center for Agricultural Research in the Dry Areas (ICARDA), P.O. Box 6299, Rabat, Morocco

**Keywords:** Markers, *Puccinia graminis* f. sp. *tritici*, QTL, GWAS, SNP

## Abstract

**Background:**

Emerging wheat stem rust races have become a major threat to global wheat production. Finding additional loci responsible for resistance to these races and incorporating them into currently cultivated varieties is the most economic and environmentally sound strategy to combat this problem. Thus, this study was aimed at characterizing the genetic diversity and identifying the genetic loci conferring resistance to the stem rust of wheat. To accomplish this, 245 elite lines introduced from the International Center for Agricultural Research in the Dry Areas (ICARDA) were evaluated under natural stem rust pressure in the field at the Debre Zeit Agricultural Research Center, Ethiopia. The single nucleotide polymorphisms (SNP) marker data was retrieved from a 15 K SNP wheat array. A mixed linear model was used to investigate the association between SNP markers and the best linear unbiased prediction (BLUP) values of the stem rust coefficient of infection (CI).

**Results:**

Phenotypic analysis revealed that 46% of the lines had a coefficient of infection (CI) in a range of 0 to 19. Genome-wide average values of 0.38, 0.20, and 0.71 were recorded for Nei’s gene diversity, polymorphism information content, and major allele frequency, respectively. A total of 46 marker-trait associations (MTAs) encompassed within eleven quantitative trait loci (QTL) were detected on chromosomes 1B, 3A, 3B, 4A, 4B, and 5A for CI. Two major QTLs with –log_10_ (p) ≥ 4 (EWYP1B.1 and EWYP1B.2) were discovered on chromosome 1B.

**Conclusions:**

This study identified several novel markers associated with stem rust resistance in wheat with the potential to facilitate durable rust resistance development through marker-assisted selection. It is recommended that the resistant wheat genotypes identified in this study be used in the national wheat breeding programs to improve stem rust resistance.

**Supplementary Information:**

The online version contains supplementary material available at 10.1186/s12863-022-01030-4.

## Background

Wheat (*Triticum aestivum* L.*)* is a leading crop, both in terms of economic value and area of production worldwide [1, 2]. Developing countries account for nearly 77% of total global wheat imports [3]. Wheat provides nearly 20% of daily world human caloric requirements [4] and demand is expected to increase to 60% by 2050 [5]. However, various challenges have hindered meeting this demand, with recurrent emerging fungal pathogens proving to be one of the leading problems worldwide [6].

Wheat stem (black) rust, caused by *Puccinia graminis* Pers. f. sp. *tritici*, Eriks. & E. Henn (*Pgt*), has been recognized as a major threat to global food security [7, 8]. Concerns regarding this disease have increased significantly, especially following the 1998 outbreak of the novel virulent race Ug99 which originated in Uganda. Since then, this race has produced 13 different variants throughout East Africa [9, 10]. The race can infect 90% of the wheat varieties grown worldwide [11] and yield losses can reach up to 100% in susceptible cultivars under conducive environmental conditions [12]. Races other than Ug99 were also reported in different parts of Western Europe. In 2013, a stem rust epidemic arose in Germany and spread to Denmark, Sweden, and the UK [13]. In 2016/2017, Italy chronicled two epidemics of wheat stem rust caused by race TTRTF, which destroyed tens of thousands of hectares of cultivated wheat [14]**.** All these reports indicate that the disease is re-emerging as a threat to wheat production globally.

Ethiopia is considered to be a hotspot for the development and evolution of new *Pgt* races [15]. Many new variants of *Pgt*, which were first identified in this country, have spread to different parts of the world. TTKSK, TKTTF, TRTTF, JRCQC, and TTTTF are the current major wheat stem races that are threatening wheat productivity in Ethiopia [16]. In 2013/2014, severe stem rust epidemics were caused by *Pgt* race TKTTF (not a member of Ug99 lineage), resulting in almost total yield loss on widely grown wheat cultivars. Since then, this race has spread widely and has been found in 10 different countries, including Western Europe [17].

To overcome this problem, host plant resistance developed through molecular marker technology is the most sustainable, cost-effective, and environmentally friendly approach for controlling rust diseases [7, 18]. Accordingly, many molecular markers linked with *Pgt* resistance were discovered throughout the wheat genome during the past couple of decades using genome-wide association mapping (GWAS). GWAS has been the most effective tool to detect several quantitative trait loci (QTLs), with moderate to minor effects against *Pgt* disease [19]. However, factors such as population structure and kinship similarity should be controlled properly to avoid false-positive QTLs. To overcome this, several models, including the mixed linear model (MLM) have been implemented. Since the first report in 2007 [20], various GWAS studies were carried out successfully and high numbers of QTLs have shown *Pgt* resistance in wheat [21–24]. So far, more than 80 genes conferring resistances to *Pgt* have been cataloged in common wheat and wheat relatives [24]. However, only a few genes are effective against all pathogen strains. Of these, *Sr2, Sr13, Sr22, Sr25, Sr26, Sr35, Sr39,* and *Sr40* were reported to be the most effective against *Ug99* [18].

The frequent co-evolution of host and pathogen remains a big challenge in the durability of the released resistant cultivars [25]. The narrow genetic diversity of cultivated wheat cultivars [22, 26] and the impact of climate change [12] are the major cause of this problem*.* Thus, additional sources of resistant QTLs, followed by marker-assisted gene pyramiding, are required to produce durable resistant varieties. Therefore, this study aimed to characterize the genetic diversity and to identify novel QTLs associated with resistance to stem rust of wheat through GWAS.

## Results

### Phenotypic variation and heritability

**Fig. 1:**
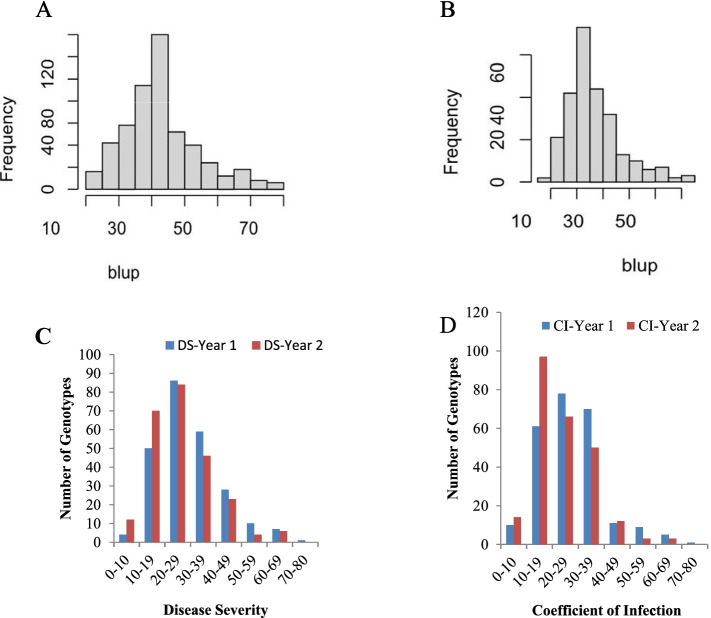
Distribution of adult plant stage disease resistance (APR) response and best linear unbiased estimates (BLUPs) as disease severity (DS), and coefficient of infection (CI). (**A**) BLUPs of DS; (**B**) BLUPs of CI; (**C**) Frequency of genotypes for disease severity (DS); (**D**) frequency of genotypes for the coefficient of infection

The performance of genotypes towards stem rust resistance varied greatly. For instance, the disease severity score was ranged between 10 and 80%. The majority (46.7%) had a disease severity (DS) score of 15–30% whilst 8.5% had a DS score of 0% (Fig. 1**,** Additional file 1). The best linear unbiased estimates (BLUP) values of DS and coefficient of infection (CI) were calculated from adjusted means of each accession across two years, and are summarized in Fig. 1.

The data of disease severity (DS) and infection response (IR) were combined to define the disease response as the coefficient of infection (CI) and 71% of lines had less than 30 (Fig. 1C). Of these, the top twenty resistance lines (presented in Table 1) ranged with the average CI values of 4.5 for pedigree SERI.1B//KAUZ/HEVO/3/AMAD/4/CHAM-6/FLORKWA-2 to 12 for pedigree SERI.1B//KAUZ/HEVO/3/AMAD/4/WEAVER/JACANA. Additional genotypes scored between 6 to 80 of CI and are presented in Additional file 1. On the other hand, all local controls (i.e. Digelu, Kubssa, Hidasse, Honqolo, and Ogolcho) were susceptible, with average CI ranging from 60 for HIDASSE to 80 for OGOLCHO and HONQOLO.

**Table 1:** Lists of top resistant lines and their pedigree during 2017/2018 main season at Debre Zeit Agricultural Research Center, Ethiopia

No	Pedigree	Disease severity and response to Sr	
		2018	2019
1	SERI.1B//KAUZ/HEVO/3/AMAD/4/CHAM-6/FLORKWA-2	10RMR	15MR
2	SERI.1B//KAUZ/HEVO/3/AMAD/4/MO88/MILAN	15MR	10MR
3	SERI.1B//KAUZ/HEVO/3/AMAD/4/TNMU/MILAN/5/WATAN-12	15MR	10MR
4	PBW343*2/KUKUN//22SAWSN – 97	10MRMS	15MR
5	SERI.1B//KAUZ/HEVO/3/AMAD/4/ESDA/SHWA//BCN	10MR	10MR
6	SERI.1B*2/3/KAUZ*2/BOW//KAUZ/4/SHIHAB-7	10MR	10MR
7	CROC-1/AE.SQUARROSA (224)//OPATA/3/FLAG-7	10MR	10MR
8	TRACHA-2/SHUHA-3/3/SHUHA-7//SERI 82/SHUHA’S′	15MRMS	10MRMS
9	SERI.1B//KAUZ/HEVO/3/AMAD/4/PFAU/MILAN	15MR	10MR
10	WATAN-7/SEKHRAH-2	10MR	15MRMS
11	WEAVER/WL 3928//SW 89.3064/3/SOMAMA-3	15MRMS	10MR
12	SERI.1B//KAUZ/HEVO/3/AMAD/4/SHUHA-7//SERI 82/SHUHA’S′	15MR	15MS
13	KAUZ’S′/SERI/3/TEVEE’S′//CROW/VEE’S′	15MRMS	15MR
14	ATTILA*2/CROW/3/VEE#5/SARA//DUCULA	15MR	15MR
15	TILILA/MUBASHIIR-1	15MR	15MR
16	QAFZAH-27/SEKSAKA-6	15MR	15MR
17	SERI.1B*2/3/KAUZ*2/BOW//KAUZ/4/SHIHAB-7	15MR	15MR
18	STAR*3/LOTUS-5/3/CHUM//7*BCN/4/FLAG-2	15MR	10MR
19	HADIAH-14/ANGI-2	10MRMS	15MRMS
20	SERI.1B//KAUZ/HEVO/3/AMAD/4/WEAVER/JACANA	15MR	15MR

The ANOVA analysis revealed highly significant variation among genotypes (*P* < 0.001) and genotype x year interactions (P < 0.001) for all parameters. Heritability (H) values for DS were 79% and IR 72%, suggesting that all parameters had a strong genetic basis. In addition, the disease distribution of the breeding lines was high between seasons, with average correlations of 0.76, 0.85, and 0.78 for DS, IT, and CI, respectively **(**Table 2**).**

**Table 2:** Mean response, variance component estimates and heritability for IR, DS, and CI variables

	DS (%)	IR(0–1)	CI
**Range**	10–80	0.3–1	3–80
**Grand mean**	33.90	0.8	28.27
**BLUEs**	32.60	0.8	26.902
**σ**^**2**^_***G***_	385.3***	0.013***	374.5***
**σ**^**2**^_**E**_	21.05*	0.000^ns^	15.7 ^ns^
**σ**^**2**^_***G*xE**_	20.70**	0.005^ns^	24.1**
**σ**^**2**^ **error**	166.10	0.004	200.3
**H**	78.95	72.2	75.7
**CV** ***r***	37.93 0.76	8.52 0.85	50.06 0.78

Disease severity (*DS*); infection response (*IR*); coefficient of infection (*CI*); *BLUEs*, best linear unbiased estimate; *σ2G* estimate of genotypic variance; *σ2E *estimate of environmental variance; *σ2GxE* is the genotype by environment interaction variance, *σ2* error is the residual error variance; heritability (*H*); r Pearson’s correlation coefficients among stem rust DS, IT, and CI between two seasons. *, **, *** and ns represents significance at *P* < 0.05, *P* < 0.01, *P* < 0.001, and not significant, respectively

### Population structure and genetic diversity analysis

**Fig. 2:**
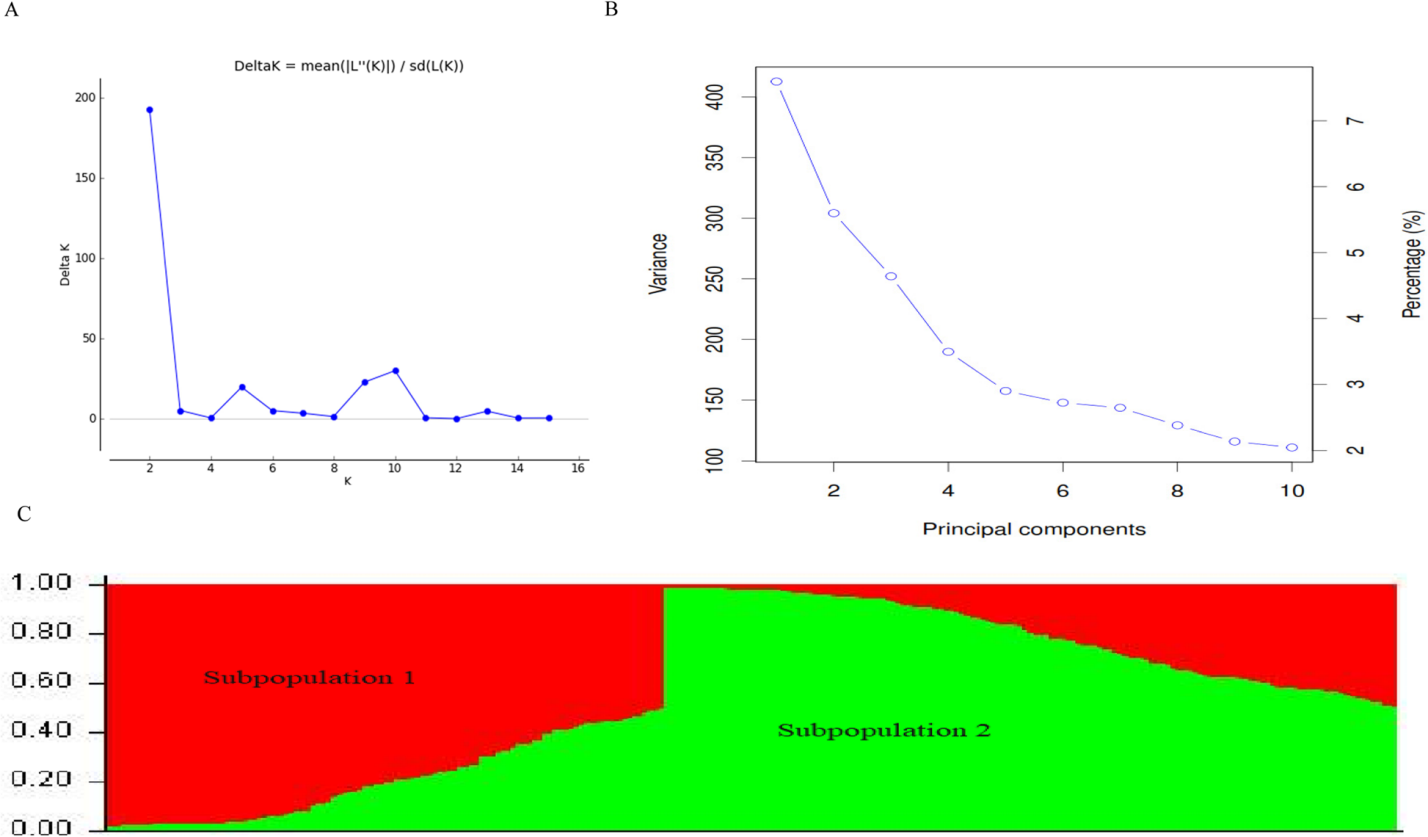
Structure clustering and principal components of 245 wheat lines based on 9523 SNP markers. (**A**) Plots of delta K; (**B**) Scree plot of PCA and (**C**) probability of population group based on K = 2

The population structure of the panel was inferred through the Bayesian clustering model, principal component analysis (PCA), and neighbor-joining (NJ) tree. The Bayesian clustering model applied on STRUCTURE software and subsequent application of STRUCTURE HARVESTER showed a delta K peak value of two (Fig. 2A). As a result, accessions were classified into two sub-populations composed of 106 and 139 lines in sub-populations 1 and 2, respectively (Fig. 2C). The scree plot of PCA showed that weak kinship existed among the lines. For the first 10 principal components (PCs), variances of SNP markers were changed from 7.5% (PC1) to 2% (PC10) and between 0 and 2% after PC10 (Fig. 2B).

**Fig. 3:**
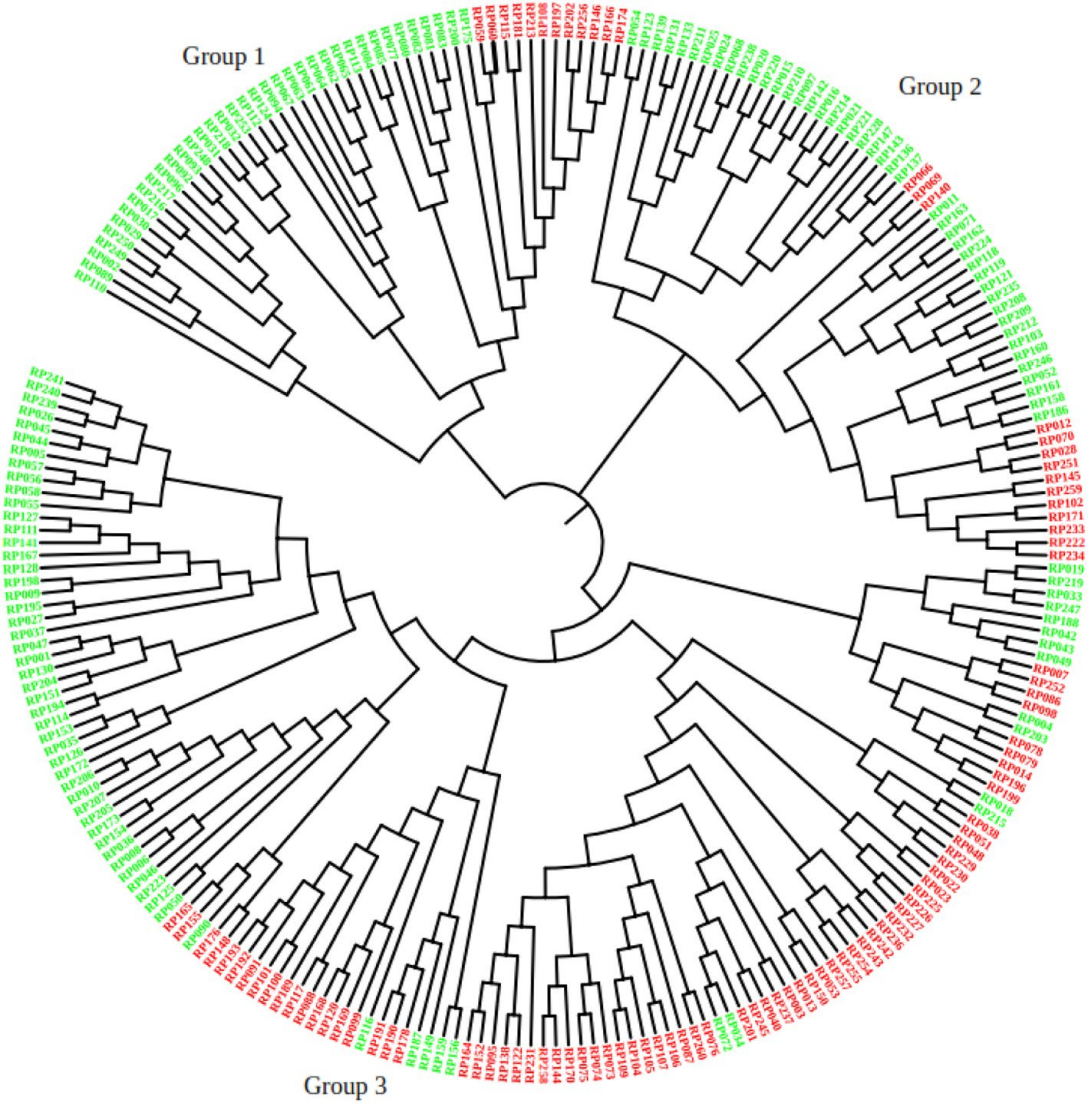
A dendrogram illustrating the clusters of wheat lines based on Nei’s genetic distance. The lines were color-coded with STRUCTURE probability distribution. Clusters with similar pedigrees and genetic backgrounds were named by their common parent

Phylogenetic tree analysis of the genetic relationship between the populations was carried out based on the distance-based neighbor-joining tree on TASSEL software v5.2.35 followed by web-based visualization software iTOL. The resulting dendrogram shows three phylogenetic groups color-coded with a STRUCTURE probability distribution. This is not consistent with the STRUCTURE result (which was two groups). Since the genotypes are elite lines, passed by complex breeding history, such inconsistency is expected. However, the majority of lines were still grouped in the same group as the STRUCTURE result and some lines were grouped in the mixed group. For instance, 78 (56%) of the lines in the first group were composed of a sub-population 1, whereas 61 lines (44%) were categorized in subpopulation 2. The second group was composed mainly of subpopulation 2, which consists of 49 (70%) lines; whereas 21 (30%) lines were classified in subpopulation 1. The third group was composed of 58 (76%) lines from subpopulation 2, and 21 (30%) lines from subpopulation 1(Fig. 3).

### Genetic data and linkage disequilibrium

**Fig. 4:**
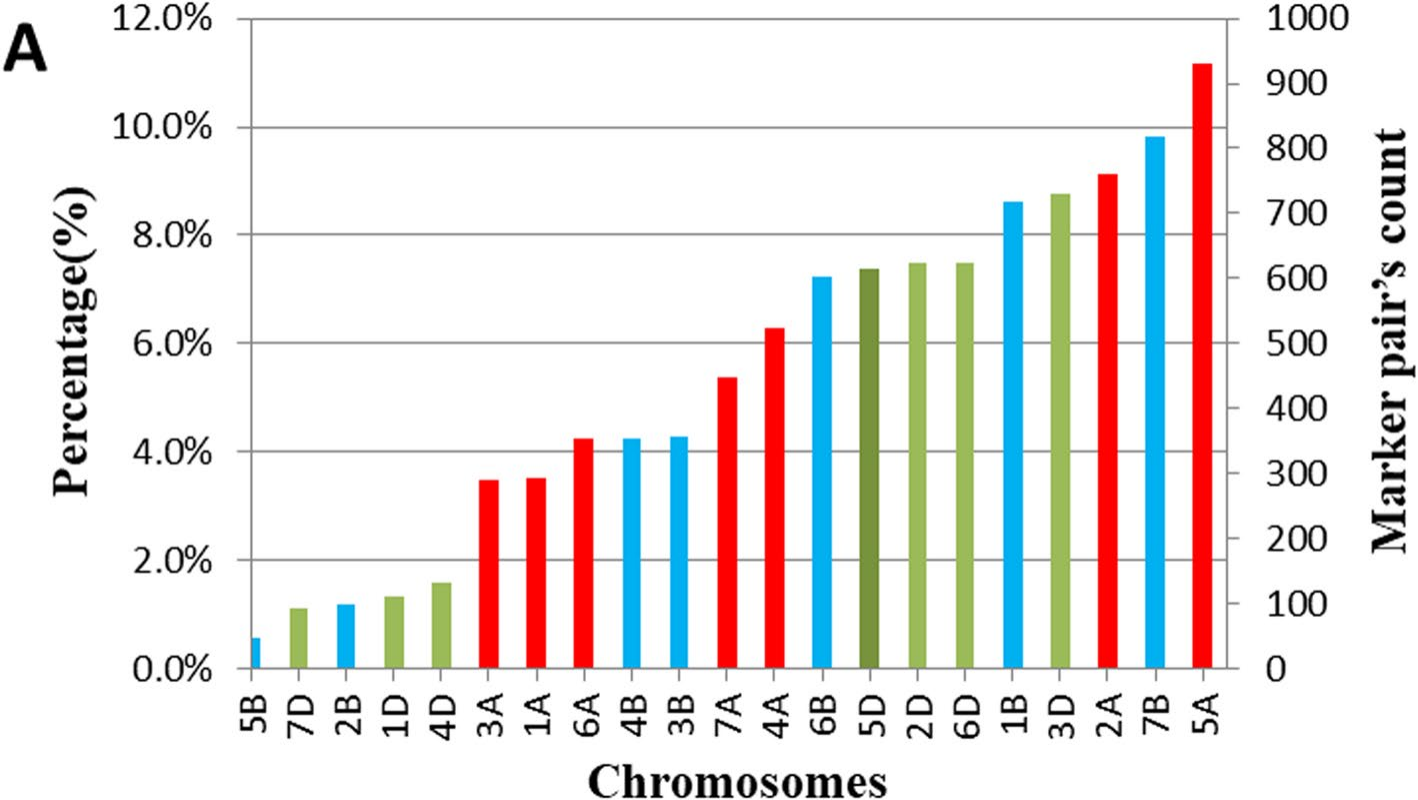
Genome-wide distributions of single nucleotide polymorphisms (SNPs) based on 15 K genotyping results

Once sub-optimal quality markers had been filtered out, 9523 SNP markers were retained from 245 lines. The distribution of SNPs across the A, B, and D sub-genomes was 50, 39, and 11%, respectively. The maximum number of SNP markers was recorded on chromosome 2B (930) and the minimum number was on chromosome 4D (48) (Fig. 4). The mean genome-wide heterozygosity, genome-wide polymorphic information content (PIC), and gene diversity were 0.006, 0.2, and 0.38, respectively. The PIC scores of SNPs varied, with only 1% being highly informative (> 0.5), while 75 and 24% of markers had moderate (0.25–0.5) and least (< 0.2) PIC scores, respectively.

**Fig. 5:**
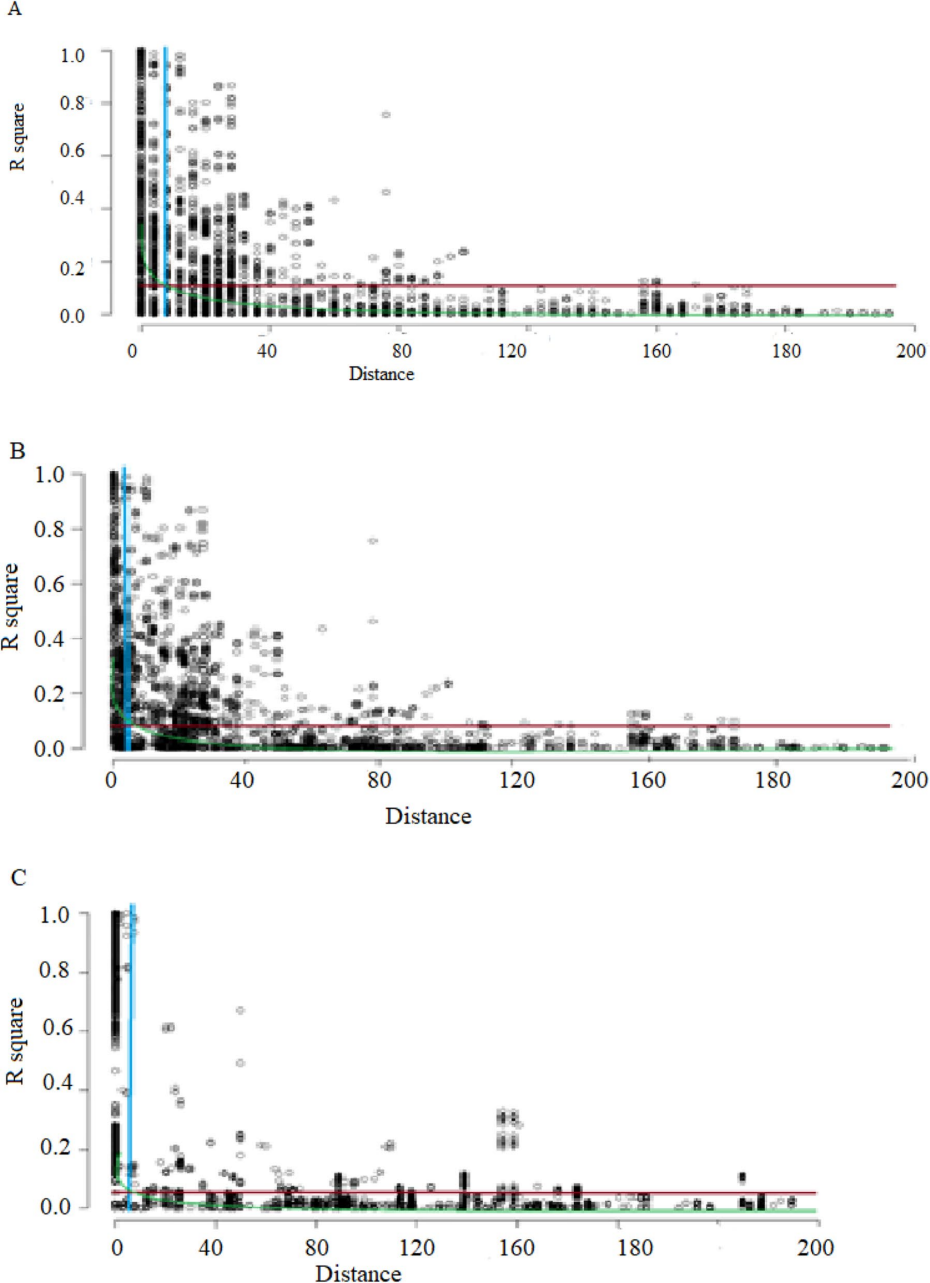
Scatterplots showing genome-wide linkage disequilibrium (LD) decays based on 15 K genotyping results in 245 wheat breeding lines. *R*^*2*^ as a function of genetic distance (cM) between pairs of SNP markers estimated for A, B, and D sub-genomes. (**A**) LD for A sub-genome; (**B**) LD of B sub-genome; (**C**) LD of D sub-genomes. The LOESS representing the decay of *R*^*2*^along genetic distance is illustrated for each genome. LD critical threshold estimated from LD distribution of pairs of unlinked SNP markers is indicated by the dashed horizontal red line

Linkage disequilibrium decay based on SNP markers of each chromosome was calculated as the Pearson correlation coefficient (r^2^) between marker pairs as a function of genetic distance (cM). The LOESS curve intercepted the line of critical value at 6 cM in A genome, 8 cM in B genome, and 5 cM in D genome, indicating that all markers within these ranges were considered as a single locus (Fig. 5).

### Marker-trait associations

A mixed linear model (MLM) was implemented for MTA, including the population structure and kinship similarity matrix (Q + K) and the BLUPs estimated values of CI of genotypes and quality checked SNP markers. The model appropriately discovered valuable MTAs with neither inflation (false-positive/type I error) nor overcorrection (false-negative/type II error) problems as depicted from the Q-Q plot (Fig. 8).

A total of 46 MTAs included in 11 QTLs were discovered for CI with the considered exploratory significant threshold (−log_1*o*_(*p*) ≥ 3). The highest number of MTAs (44) was detected on the B sub-genome, of which 36, 4, 3, and 1 MTAs were located on chromosomes 1B, 3B, 4B, and 5B, respectively (Table 3**,** Fig. 7). The remaining 2 QTLs were identified from chromosomes 3A and 4A. The explained phenotypic variance of QTLs was taken from the most significant SNP marker (QTL tag-SNP). The three QTLs with high explained phenotypic variance (PV) of tag SNPs were EWYP1B.4 (8.8%), EWYP1B.5 (8.37%), and EWYP1B.3 (8.16%). The other PV of QTLs tag-SNPs ranged from 7.48 to 4.76% (Table 3**,** Fig. 7). Details of markers distribution in each accession are presented in Additional file 1.

**Table 3:** Lists of QTLs identified for adult plant resistance (APR) to wheat stem rust

QTLs	SNPs	Markers Name	Alleles	Chr^**a**^	Pos^**b**^	Pos^**c**^	MAF	–log_**10**_P	R^**2**^	FDR^**d**^	***Sr*** genes	References
EWYP1B.1	IWA6489.1	wsnp_Ku_c13229_21142792	T/C	1B	30.34	unknown	0.213439	4.398146	7.14	*0.0192*	*Sr31*	Mettin et al.*,* 1973 [27]*;* Zeller*,* 1973 [28]
	IWB29508	Excalibur_c95327_51	A/G	1B	43.86	9.37986	0.418972	3.000182	5.91	0.0265		
EWYP1B.2	IWB29475	Excalibur_c94756_540	T/C	1B	57.6	unknown	0.41502	3.398788	6.63	0.0163		new
	IWB1569	BobWhite_c22266_315	C/T	1B	60.62	329.48968	0.213439	4.17831	8.16	0.0050		
	IWB43258	Kukri_c26168_423	C/A	1B	60.62	1.417758	0.462451	3.07204	6.06	0.0171		
EWYP1B.3	IWB23446	Excalibur_c20228_135	A/G	1B	64.1	305.2701	0.094862	3.717446	7.27	0.0130		new
	IWB54643	RAC875_c18282_1390	T/C	1B	64.1	unknown	0.090909	3.676459	7.15	0.0171		
	IWA5592	wsnp_Ex_rep_c69266_68192766	T/C	1B	64.1	299.97785	0.094862	3.668026	7.13	0.0130		
	IWB6405	BS00011450_51	T/C	1B	64.1	318.23318	0.13834	3.473183	6.74	0.0080		
	IWA131	wsnp_BE443531B_Ta_1_1	C/T	1B	64.32	156.68751	0.114625	3.490435	6.77	0.0231		
	IWB48689	Kukri_rep_c101799_95	C/A	1B	64.46	unknown	0.114625	3.490435	6.77	0.0231		
	IWA3631	wsnp_Ex_c38116_45719983	C/T	1B	64.89	340.37296	0.079051	3.957779	7.72	0.0775		
	IWB51549	Ra_c23839_884	T/C	1B	65.42	367.44011	0.146245	3.61409	7.02	0.0080		
	IWB31732	GENE-0193_197	A/G	1B	65.42	367.44095	0.146245	3.471958	6.74	0.0080		
	IWA6890	wsnp_Ku_c30982_40765254	T/G	1B	66.07	336.20988	0.075099	4.081555	7.97	0.1850		
	IWB58051	RAC875_c44575_561	C/T	1B	66.07	325.79901	0.075099	3.973875	8.40	0.0266		
	IWB461	BobWhite_c1318_691	T/C	1B	66.73	387.64464	0.150198	3.618488	7.03	0.0080		
	IWB37720	JD_c64600_281	G/A	1B	67.14	409.62805	0.893281	3.718149	7.48	0.0208		
	IWA106	wsnp_BE442716B_Ta_2_1	T/G	1B	67.14	403,156,221	0.102767	3.67975	7.15	0.0208		
	IWB38394	Ku_c13515_171	C/T	1B	67.14	unknown	0.102767	3.67975	7.15	0.0208		
	IWB35871	IACX2701	C/T	1B	67.38	413.08886	0.098814	3.795799	7.43	0.0208		
	IWB10444	BS00070139_51	A/C	1B	68.04	418.16246	0.150198	3.618055	7.03	0.0080		
	IWB27852	Excalibur_c59016_839	A/G	1B	68.04	426.07288	0.150198	3.618055	7.03	0.0080		
	IWB56778	RAC875_c32894_1038	C/T	1B	68.04	unknown	0.150198	3.354313	8.83	0.0080		
	IWB59327	RAC875_c5796_424	A/G	1B	68.04	IWB59327	0.150198	3.618055	7.03	0.0080		
	IWB8148	BS00038929_51	T/C	1B	68.04	417.85674	0.150198	3.618055	7.03	0.0080		
EWYP1B.4	IWB74145	tplb0023b14_704	G:T	1B	70.08	438.2896	0.339921	3.35714	6.59	0.0245		new
	IWB47566	Kukri_c73734_175	C/T	1B	76.89	532,565,453	0.213439	4.268153	8.37	0.0282		
	IWB60433	RAC875_c7674_634	G/A	1B	76.89	532.56545	0.27668	3.545613	7.40	0.0282		
	IWB6504	BS00011973_51	T/G	1B	76.89	531,855,670	0.280632	3.485333	6.78	0.0282		
	IWA775	wsnp_CAP11_c543_375403	A/G	1B	76.89	542.43163	0.217391	3.044683	5.89	0.0282		
	IWA5228	wsnp_Ex_rep_c66389_64588992	A/G	1B	79.77	561.70458	0.110672	3.731492	7.28	0.0080		
	IWB74900	tplb0048b10_1365	A/G	1B	79.77	552.5332	0.110672	3.731492	7.28	0.0080		
EWYP1B.5	IWB10621	BS00072791_51	A/G	1B	105.83	629.26299	0.27668	3.778586	7.61	0.0080		new
	IWB70380	Tdurum_contig32775_78	A/G	1B	112.07	unknown	0.335968	3.732007	7.26	0.0080		
	IWB66198	Tdurum_contig10036_977	C/A	1B	114.13	634.6536	0.094862	3.49178	6.81	0.0080		
EWYP3A	IWB73429	Tdurum_contig777_260	G/A	3A	20.74	102.20749	0.339921	3.609065	7.12	*0.0080*	*Sr27*	McIntosh et al.*,* 1995 [29]
EWYP3B.1	IWB67769	Tdurum_contig12899_342	T/C	3B	9.7	5.585837	0.379447	3.484232	6.90	*0.0210*	*Sr2*	Ausemus et al.*,* 1946 [30]; Knott, 1968 [31]
	IWB23457	Excalibur_c20277_483	A/G	3B	9.7	5.58572	0.371542	3.042077	5.88	*0.0210*		
	IWB67389	Tdurum_contig12008_803	T/C	3B	9.7	5.584656	0.395257	3.016518	5.83	0.0210		
EWYP3B.2	IWB75222	tplb0059m03_622	C/T	3B	20.14	7.373615	0.434783	3.022107	5.95	0.0208		new
EWYP4A	IWB72664	Tdurum_contig59603_74	G/A	4A	26.5	9.92939	0.150198	3.240453	6.40	0.260		new
EWYP4B	IWB24798	Excalibur_c29127_552	G/A	4B	64.58	232.88033	0.094862	3.73037	7.26	0.0208		new
	IWB48189	Kukri_c8973_1986	T/C	4B	62.22	Unknown	0.098814	3.126412	4.76	0.073		
	IWB53758	RAC875_c13639_2159	T/C	4B	62.92	272.17459	0.098814	3.126412	4.76	0.0190		
EWYP5B	IWB56412	RAC875_c30011_426	C/T	5B	104.55	571.47521	7.08548	2.9914	5.77	*0.0210*	*Sr56*	Park 2016 [32]; Yu et al.*,* 2014 b[33]

*QTLs* Quantitative trait loci, *SNPs* Single nucleotide polymorphism, *Chr*^*a*^ Chromosome position, *Pos*^*b*^ Marker’s genetic position mapped in the wheat 90KSNP consensus map [34] in centimorgans (cM); Pos^c^, marker’s physical position produced by the International Wheat Genome Sequencing Consortium (IWGSC RefSeq v1.0 )[35] in megabase pairs (Mbp); FDR^d^, The false-discovery rate adjusted *P*-values; (MAF), minor allele frequency; R^2^, phenotypic variance explained by the markers

## Discussion

Stem rust has been increasing in severity and incidence and now poses a serious threat to global wheat production [8]**.** To overcome this threat, efforts are ongoing worldwide to monitor rust diseases, identify rust pathotypes, and evaluate wheat germplasm for rust resistance [36]. As part of the global effort, this study was designed to quantify the existing allelic variation of breeding lines and to search for sources of resistant QTL for *Pgt* resistance*.* Consequently, 245 elite bread wheat lines were evaluated in the field condition to identify QTLs for adult plant resistance to wheat stem rust. A significant variation was observed between breeding lines for adult plants’ resistance to stem rust. This study detected several MTAs included in 11 different QTLs with different effects that could potentially play an important role in future marker-assisted pyramiding against the disease.

### Field evaluation of wheat germplasm for resistance to stem rust

Disease response characterization under high disease pressure in field conditions remains the best stem rust management strategy in breeding for developing stem rust-resistant cultivars [37]. Ethiopia is considered to be a hotspot for the development of *Pgt* race diversity and frequent disease epidemics. Studies carried out in Ethiopia showed that most previously identified races such as TTKSK, TKTTF, TTTTF, TRTTF, RRTTF, and others were virulent on most varieties grown in the country [38]. Accordingly, many field evaluation studies for *Pgt* response have been carried out in different wheat-growing regions of the country [22, 39, 40]. Most elite breeding lines skewed towards moderate resistance, although some differences were observed between individual genotypes. The two parameters (i.e. DS and IR) showed moderate to high heritability with significant variation among lines and genotype x year interactions, indicating that most of the existing variation was due to genetic bases. CI has been used as the most efficient trait to discover QTLs of stem rust resistance in wheat via GWAS analysis [23, 41].

### Population structure and genetic diversity

Systematic characterization of population structure and genetic diversity provides a foundation for efficient exploitation of genetic resources and can enhance breeding for durable stem rust resistance in wheat. For the population structure study, three different approaches were applied. Although there is considerable overlap between the three techniques for population analysis, the overall conclusion suggests that there is no clear and substantial separation between individual genotypes. This could be owing to the panel’s complicated evolutionary and breeding history. It is suggested that more researches need to be done to better understand the relationships between genotypes from different groups.

The mean PIC and gene diversity of the genome was 0.25 and 0.3, respectively. Several studies have previously reported various rates of Nei’s gene diversity and PIC in different wheat populations [22, 34, 42–44].

### Linkage disequilibrium and MTAs

**Fig. 6:**
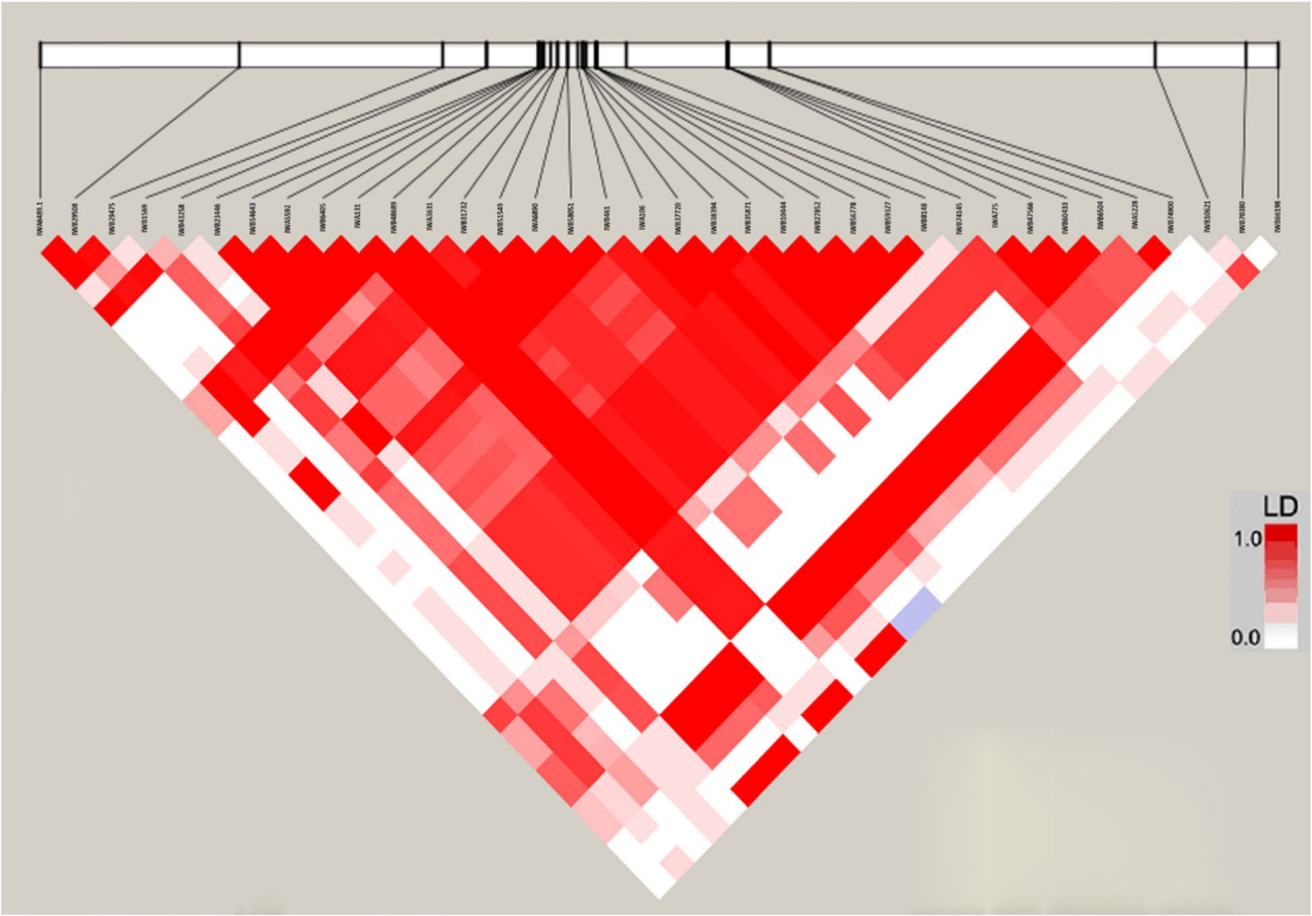
The linkage disequilibrium blocks formed by the 36 significantly associated SNPs with APR to stem rust on chromosome 1B

The LD of the genome and sub-genomes of the current panel was estimated using SNP markers. The fastest LD decay was observed on the D sub-genome, which agreed with the previous report [45]. The LOESS curve intercepted the line of critical value at 6 cM in A genome, at 8 cM in B genome, and 5 cM in the D genome, indicating that all markers within these ranges are considered as a single locus (Fig. 5). Since many significant SNP markers (36 MTAs) were identified in the present study, the LD pattern in chromosome 1B was analyzed independently and detected five LD blocks **(**Fig. 6**)**. Similar large LD blocks in 1B chromosomes have been reported previously [46].

**Fig. 7:**
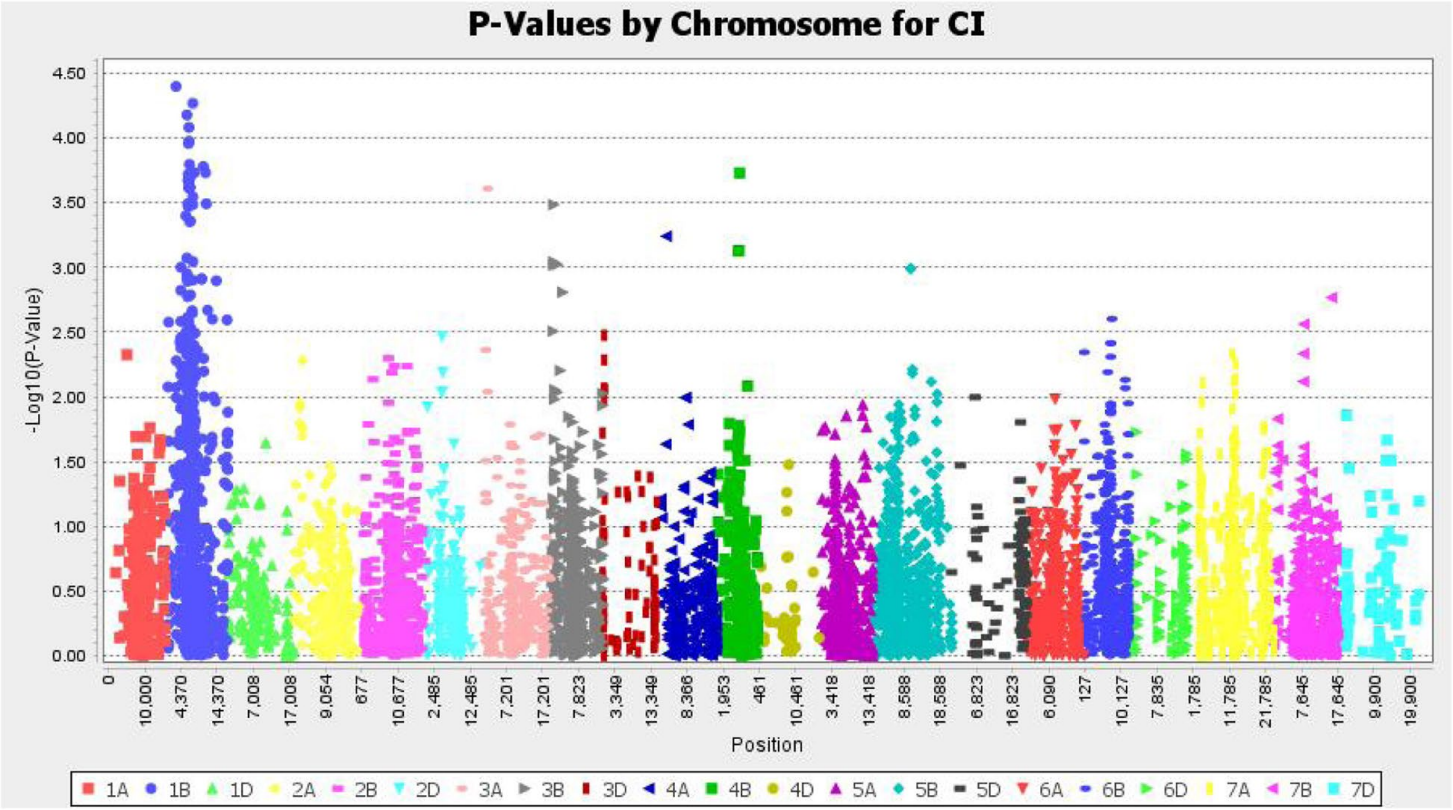
GWAS results of the Manhattan plot along with the 21 chromosomes showing significantly associated markers with adult plant stem rust resistance. The position of each marker was based on the wheat consensus SNP map [34]

**Fig. 8:**
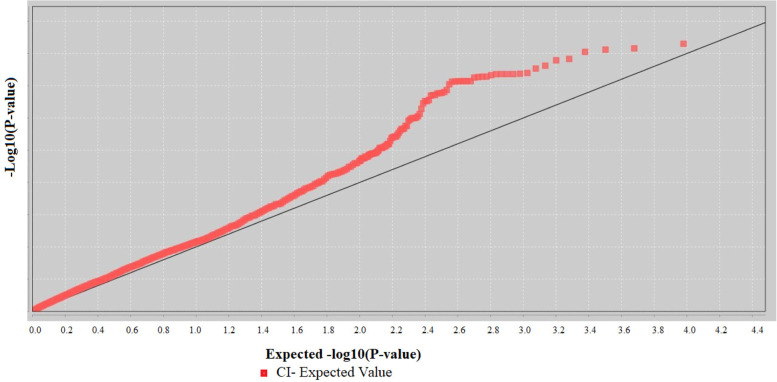
Q-Q plot for stem rust resistance in a panel of 245 wheat breeding lines using the MLM model. The plots show the observed *p*-values (*p*) for the association between CI and each tested marker expressed as –log 10 (*P*-value) of *p* (y-axis) plotted against –log_10_ *P* of the expected p-values (x-axis) under the null hypothesis of no association for the analyses

The current study unveiled 46 SNP significant markers encompassed within 11 QTLs. Of these, only two QTLs (EWYP1B.1, EWYP1B.2) were identified as major QTLs (−log_1*o*_(*p*) ≥ 4). EWYP1B.1, EWYP3A, EWYP3B.1, and EWYP5B, respectively, were found near genomic areas of *Sr31, Sr27, Sr2,* and *Sr56* [33]. The remaining seven QTLs (EWYP1B.2, EWYP1B.3, EWYP1B.4, EWYP1B.5, EWYP3B.2, EWYP4A, and EWYP4B) were newly discovered in the current study. These new QTLs could play paramount importance in enhancing *Pgt* resistance through marker-assisted selection or introgression.

We found five QTLs on chromosome 1B (EWYP1B.1, EWYP1B.2, EWYP1B.3, EWYP1B.4, and EWYP1B.5) that encompassed 36 MTAs ranging in size from 30.34 cM (*wsnp_Ku_c13229_21142792*) to 114 cM (*Tdurum_contig10036_977*). On this chromosome, three resistance genes (*Sr14, Sr31,* and *Sr58*) were cataloged previously [32]. Of these, only *Sr31* has been reported in association with wheat stem rust disease at locus EWYP1B. 1[27, 28]. The remaining four QTLs (EWYP1B.2, EWYP1B.3, EWYP1B.4, and EWYP1B.5) were likely novel resistance loci identified in the current study. Four of the 36 MTAs found on this chromosome have previously been associated to other wheat diseases: *snp_BE442716B_Ta_2_1* and *wsnp_Ex_rep_c69266_68192766* with stripe rust [47], *wsnp_Ex_c38116_45719983* with Fusarium head blight [48], and *BS00070139_51* with crown rot resistance [49].

On chromosomes 3A, 3B, 4A, 4B, and 5B, the additional six QTLs containing 10 MTAs were discovered. The marker *Tdurum_contig777_260* (IWB73429) designated as EWYP3A QTL was adjacent to the all-stage resistance gene *Sr27* which is transferred from *Secale cereale* and *Sr35* gene which is transferred from *Triticum monococcum* [32]. Because EWYP3A is so close to the *Sr27* gene area, *Sr27* is most likely the underlying gene for this region. On the short arm of chromosome 3B, *Sr2* came from *Triticum dicoccum* and *Sr12* originated from *Triticum turgidum* ssp. were cataloged previously [32]. On this chromosomal, we discovered the EWYP3B.1 QTL, which consists of three markers (*Tdurum_contig12899_342, Excalibur_c20277_483,* and *Tdurum_contig12008_803*). The nearest *Sr* gene to these markers was the *Sr2* gene [30, 31]*.* This *Sr2* gene has been extensively used in breeding as a source of durable and broad-spectrum adult plant resistance. Individual genotypes carrying the favorable allele of these SNP markers have shown an apparent difference in the CI score (Additional file 1). On chromosome 5B, the SNP marker *RAC875_c30011_426* (*IWB56412*) explained 5.7% of the total phenotypic variation. This marker is found near the chromosome region previously discovered the *Sr56* gene that confers the APR to wheat stem rust [32, 33]. To the best of our knowledge, the other three QTLs, EWYP3B.2, EWYP4A, and EWYP4B, which were found on chromosomes 3B, 4A, and 4B, respectively, have never been reported and could potentially be novel QTL sources for stem rust resistance breeding programs.

## Conclusions

This study characterized the genetic diversity of elite ICARDA breeding lines and performed GWAS based on the evaluation of field stem rust. As a result, substantial genetic variability and field disease response to *Pgt* was observed among the lines. The study detected several potentially novel loci associated with *Pgt* resistance. These markers could provide useful genetic information to unlock the genetic basis of resistance to *Pgt* in wheat. Furthermore, the result will accelerate the introgression of identified resistance QTLs in the wheat breeding program through marker-assisted introgression. The identified resistant lines could also be used as crossing parents in stem rust-resistant breeding programs.

## Materials and methods

### Plant materials, field stem rust trials, and disease pathotyping

A set of 245 elite breeding lines was obtained from the International Center for Agricultural Research in the Dry Areas (ICARDA) shuttle breeding program. Field screening was conducted in Ethiopia for two consecutive cropping seasons (2018 and 2019) at the Debre Zeit Agricultural Research Center (DARC). DARC is located at 08° 44′ N latitude and 38° 58′ E longitude and 1900 m.a.s.l with 19 °C annual average temperature and 851 mm rainfall. The experiment was conducted using an augmented design, including five local cultivars (Digelu, Kubssa, Hidasse, Honqolo, and Ogolcho) as checks. Each line was planted in a 1 m long single row and the distance between rows was 30 cm. The border of each block was surrounded by susceptible local spreader wheat varieties to promote natural stem rust infection.

Stem rust phenotyping was conducted based on disease severity (DS) and infection response (IR) under natural disease pressure [50]. Both parameters were recorded three times for each line in each year. The highest recorded value was then taken for the GWAS analysis after calculating the coefficient of infection (CI) from the two parameters (i.e. DS and IR).

The CI was calculated by multiplying the DS by a constant value of IR recorded according to Yu et al. (2011). IR values were recorded with the following scale: immune (I) = 0.0, R (resistant) = 0.2, resistant to moderately resistant (RMR) = 0.3, moderately resistant (MR) = 0.4, moderately resistant to moderately susceptible (MRMS) = 0.6, moderately susceptible (MS) = 0.8, moderately susceptible to susceptible (MSS) = 0.9 and susceptible (S) = 1.0.

### Statistical analysis of phenotypic data

Analysis of variance (ANOVA) was performed for DS, IR, and CI using the *nlme* package in the R 4.0.2 environment (Pinheiro et al., 2020) fitting the value of DS, IR, and CI as a function of lines, years, and a combination of lines and years. To determine the consistency of DS, IR, and CI, Pearson correlation coefficients between seasons were calculated.

Broad-sense heritability (*H* ^*2*^) was calculated using the following formula:

H ^2^**=** $$\frac{\upsigma^2\ {G}}{\upsigma^2\ {G}+\left({\upsigma}^2{G} XE\right)/n+\left(\ {\upsigma}^2\ error\right)/n}$$

Where σ^2^_G_ is the genotypic variance, σ^2^_E_ is the environmental variance, σ^2^_GXE_ is the genotype by environment interaction variance, σ^2^ error is the residual error variance and *n* is the number of years.

To reduce false-positive associations, best linear unbiased predictors (BLUPs) for CI were calculated using a mixed model in *lme4* package implemented in R environment [51] according to the following model where *y* is the response variable:

$$y= lmer\ \left( Trait\sim \left(\ 1| Genetype\right)+\left(1| Year\right)\right)$$

### Population structure and genetic diversity

The optimal sub-populations of the panel were estimated based on three different approaches. The Bayesian model-based population structure was estimated from 100 unlinked SNP markers located at least 10 cM apart across the genome using STRUCTURE 2.3.1 software [52, 53]. To execute this, three independent runs were performed for each hypothetical K value run from 2 to 15 with the length of the burn-in period of 10,000 steps followed by 100,000 Monte Carlo Markov Chain (MCMC). The results obtained from this procedure were used in a web-based informatics tool namely, “Structure Harvester” [54] to define the optimal K value, based on ∆K method Evanno, 2005 [55]. Each genotype was assigned to one subpopulation based on its membership probability. The second approach used to determine the optimal subpopulation was based on a marker-based kinship matrix (K matrix) on a scaled identity-by-state method using the whole set of SNP markers from TASSEL 5 software [56]. Finally, the principal components analysis (PCA) of genetic relatedness was performed with the same software and added to the regression model as a covariant.

Genetic diversity was estimated based on polymorphic information content (PIC), heterozygosity, and Nei’s gene diversity using the whole set of SNP markers from PowerMarker 3.25 software [57]. Phylogenetic analysis based on distance-based neighbor-joining method was calculated with TASSEL 5 software and visualized through web-based program iTOL (v 4.3.2) [58].

### Genotyping, linkage disequilibrium, and genome-wide association analysis

DNA extraction of lines was carried out on one-week-old seedlings following the protocol described by Allen et al. (2006) [59] using Cetyeltrimethylammonium bromide (CTAB). Genotyping was performed by Illumina iSelect 15 K single nucleotide polymorphism (SNP) wheat array and called by GenomeStudio V2011.1 software. The resulting 13,006 SNPs were further screened using those only minor allelic frequency (MAF) > 5%, and missing data percentage of < 10%. Five lines were excluded as a result of this screening. Finally, 9523 quality SNP markers were generated from 245 lines that were used for further analysis.

The resulting SNP data were subjected to linkage disequilibrium (LD) analysis as squared allelic frequency correlations (R^2^) between each pair implemented in TASSEL v5.2 and GAPIT (Genomic Association and Prediction Integrated Tool) R package [60]. The critical R^2^ value (where the LD is due to the physical linkage) was determined by taking the 95% of R^2^ data of unlinked markers as the threshold, according to Breseghello and Sorrells (2006) [61].

Marker-trait association analysis (MTAs) between the BLUP value of CI and SNPs markers were analyzed using a mixed linear model (MLM) in TASSEL 5.2 software. Using the formula: y = Xα + Qδ + Kμ + e; where y = phenotypic values, X is SNP marker genotypes, α is a vector containing fixed effects as a result of the genotype, Q is population structure as PCA, **δ** is a vector containing fixed effects resulting from population structure, K is the relative kinship matrix, μ is a vector of random additive genetic effects and e is a vector of residuals. Marker trait associations were declared significant at a threshold value of –log_10_ (p) ≥ 3 (corresponding *p value* ≤ 0.001) [62].

## Supplementary Information

**Additional file 1:** List of pedigree stem rust response and associated SNPs for wheat stem rust.

## Data Availability

The datasets generated and/or analyzed during the current study are available in the figshare data repository, 10.6084/m9.figshare.17711150.v3.

## Abbreviations

ANOVA: Analysis of variance
BLUP: Best linear unbiased prediction
CI: Coefficient of infection
DARC: Debre Zeit Agricultural Research Center
GWAS: Genome-wide association study
DS: Disease severity
ICARDA: International Center for Agricultural Research in the Dry Areas
IR: Infection response
LD: Linkage disequilibrium
MAF: Minor allele frequency
MAS: Marker-assisted selection
MCMC: Markov chain Monte Carlo
MLM: Mixed Linear Model
MTA: Marker-trait association
PC: Principal components
PCA: Principal component analysis
*Pgt*: Wheat stem rust fungus *Puccinia graminis*
PIC: Polymorphism information content
PV: Phenotypic variance
QQ: Quantile-quantile
QTL: Quantitative trait locus
SNP: Single nucleotide polymorphism

## References

[1] Giraldo P, Benavente E, Manzano-Agugliaro F, Gimenez E (2019). Worldwide research trends on wheat and barley: A bibliometric comparative analysis. Agronomy.

[2] Marocco E, Milo M. Food Outlook. 2019.

[3] Enghiad A, Ufer D, Countryman AM, Thilmany DD. An Overview of Global Wheat Market Fundamentals in an Era of Climate Concerns. Int. J Agron. 2017;2017.

[4] Lucas H (2012). Breakout session P1.1 National Food Security-The Wheat Initiative-an International Research Initiative for Wheat Improvement. Second Glob Conf Agric Res Dev..

[5] Schütz H, Jansen M, Verhoff MA (2011). Vom alkohol zum liquid ecstasy (GHB) - Ein überblick über alte und moderne K.-o.-Mittel - Teil 3: γ-Hydroxybuttersäure (GHB, “liquid ecstasy”). Arch Kriminol..

[6] Figueroa M, Hammond-Kosack KE, Solomon PS (2018). A review of wheat diseases—a field perspective. Mol Plant Pathol..

[7] Gao L, Rouse MN, Mihalyov PD, Bulli P, Pumphrey MO, Anderson JA (2017). Genetic characterization of stem rust resistance in a global spring wheat germplasm collection. Crop Sci..

[8] Long L, Yao F, Yu C, Ye X, Cheng Y, Wang Y (2019). Genome-Wide Association Study for Adult-Plant Resistance to Stripe Rust in Chinese Wheat Landraces (*Triticum aestivum* L.) From the Yellow and Huai River Valleys. Front. Plant Sci..

[9] Fetch T, Zegeye T, Park RF, Hodson D, Wanyera R (2016). Detection of wheat stem rust races TTHSK and PTKTK in the Ug99 race group in Kenya in 2014. Plant Dis..

[10] Newcomb M, Olivera PD, Rouse MN, Szabo LJ, Johnson J, Gale S, et al. Kenyan isolates of *Puccinia graminis* f. sp. *tritici* from 2008 to 2014: Virulence to SrTmp in the Ug99 race group and implications for breeding programs. Phytopathology. 2016(106):729–36. 10.1094/PHYTO-12-15-0337-R 27019064

[11] Singh RP, Hodson DP, Huerta-Espino J, Jin Y, Bhavani S, Njau P (2011). The emergence of Ug99 races of the stem rust fungus is a threat to world wheat production. Annu Rev Phytopathol..

[12] Prank M, Kenaley SC, Bergstrom GC, Acevedo M, Mahowald NM. Climate change impacts the spread potential of wheat stem rust, a significant crop disease. Environ Res Lett. 2019;14.

[13] Saunders DGO, Pretorius ZA, Hovmøller MS (2019). Tackling the re-emergence of wheat stem rust in Western Europe. Commun Biol..

[14] Olivera PD, Sikharulidze Z, Dumbadze R, Szabo LJ, Newcomb M, Natsarishvili K (2019). Presence of a Sexual Population of *Puccinia graminis* f. sp. *tritici* in Georgia Provides a Hotspot for Genotypic and Phenotypic Diversity. Phytopathology®..

[15] Leppik EE (1970). Gene Centers of Plants as Sources of Disease Resistance. Annu Rev Phytopathol..

[16] Hundie B. Evaluation of Advanced Bread Wheat Lines for Field and Seedling Resistance to Stem Rust (<i>*Puccinia graminis*</i> f. sp. <i>tritici</i>). Am J Biol Environ Stat. 2018;4:74.

[17] Rahmatov M, Pretorius ZA, Bhavani S. Sources of Stem Rust Resistance in Wheat-Alien Introgression Lines. 2016. 10.1094/PDIS-12-15-1448-RE. 10.1094/PDIS-12-15-1448-RE 30682285

[18] Aktar-Uz-Zaman M, Tuhina-Khatun M, Hanafi MM, Sahebi M (2017). Genetic analysis of rust resistance genes in global wheat cultivars: an overview. Biotechnol Biotechnol Equip..

[19] Alqudah AM, Sallam A, Stephen Baenziger P, Börner A (2020). GWAS: Fast-forwarding gene identification and characterization in temperate Cereals: lessons from Barley – A review. J Adv Res.

[20] Crossa J, Burgueño J, Dreisigacker S, Vargas M, Herrera-Foessel SA, Lillemo M (2007). Association analysis of historical bread wheat germplasm using additive genetic covariance of relatives and population structure. Genetics..

[21] Yu LX, Lorenz A, Rutkoski J, Singh RP, Bhavani S, Huerta-Espino J (2011). Association mapping and gene-gene interaction for stem rust resistance in CIMMYT spring wheat germplasm. Theor Appl Genet..

[22] Muleta KT, Rouse MN, Rynearson S, Chen X, Buta BG, Pumphrey MO (2017). Characterization of molecular diversity and genome-wide mapping of loci associated with resistance to stripe rust and stem rust in Ethiopian bread wheat accessions. BMC Plant Biol..

[23] Gao L, Turner MK, Chao S, Kolmer J, Anderson JA (2016). Genome Wide Association Study of Seedling and Adult Plant Leaf Rust Resistance in Elite Spring Wheat Breeding Lines. PLoS One..

[24] Leonova IN, Skolotneva ES, Orlova EA, Orlovskaya OA, Salina EA (2020). Detection of genomic regions associated with resistance to stem rust in Russian spring wheat varieties and breeding germplasm. Int J Mol Sci..

[25] Tadesse W, Halila H, Jamal M, Hanafi S, Assefa S, Oweis T (2017). Role of Sustainable Wheat Production to Ensure Food Security in the CWANA region. J Exp Biol Agric Sci..

[26] Novoselović D, Bentley AR, Šimek R, Dvojković K, Sorrells ME, Gosman N (2016). Characterizing Croatian wheat germplasm diversity and structure in a European context by DArT markers. Front Plant Sci.

[27] Mettin D, Bluthner WD, Schlegel G. Additional evidence on spontaneous 1B/1R wheat-rye substitutions and translocations. Proc fourth Int wheat Genet Symp Alien Genet Mater. 1973;4:179–84.

[28] Zeller FJ. 1B/1R wheat-rye chromosome substitutions and translocations. Proc fourth Int wheat Genet Symp Alien Genet Mater. 1973;4:209–21.

[29] McIntosh RA, Wellings CR, Park R (1996). Wheat Rusts: An Atlas of Resistance Genes. Australas Plant Pathol..

[30] Ausemus ER, Harrington JB, Reitz LP, Worzella WW (1946). A summary of genetic studies in hexaploid and tetraploid wheats. J Am Soc Agronomy.

[31] Knott DR (1968). The inheritance of resistance to stem rust races 56 and 15B-1L (Can.) in the wheat varieties Hope and H-44. Can J Genet Cytol.

[32] Park RF. Wheat: Biotrophic Pathogen Resistance. In: Reference Module in Food Science. Amsterdam: Elsevier; 2016.

[33] Yu LX, Barbier H, Rouse MN, Singh S, Singh RP, Bhavani S (2014). A consensus map for Ug99 stem rust resistance loci in wheat. Theor Appl Genet..

[34] Wang S, Wong D, Forrest K, Allen A, Chao S, Huang BE (2014). Characterization of polyploid wheat genomic diversity using a high-density 90 000 single nucleotide polymorphism array. Plant Biotechnol J..

[35] Appels R, Eversole K, Feuillet C, Keller B, Rogers J, Stein N (2018). Shifting the limits in wheat research and breeding using a fully annotated reference genome. Science..

[36] Bhardwaj SC, Singh GP, Gangwar OP, Prasad P, Kumar S (2019). Status of wheat rust research and progress in rust management-Indian context. Agronomy..

[37] Singh RP, Hodson DP, Jin Y, Lagudah ES, Ayliffe MA, Bhavani S (2015). Emergence and spread of new races of wheat stem rust fungus: Continued threat to food security and prospects of genetic control. Phytopathology..

[38] Mitiku M, Bacha Hei N, Abera M (2018). Characterization of Slow Rusting Resistance Against Stem Rust (*Puccinia graminis* f. sp. *tritici*) in Selected Bread Wheat Cultivars of Ethiopia. Adv Crop Sci Technol.

[39] Abebe T, Dawit W, Woldeab G (2013). Physiological Races and Virulence Diversity of *Puccinia graminis* pers. f. sp. *tritici* eriks. & e. Henn. on Wheat in Tigray Region of Ethiopia. Int J Phytopathol..

[40] Hei N, Shimelis HA, Laing M, Admassu B (2015). Assessment of Ethiopian wheat lines for slow rusting resistance to stem rust of wheat caused by *Puccinia graminis* f.sp. *tritici*. J Phytopathol..

[41] Yu L-X, Morgounov A, Wanyera R, Keser M, Singh SK, Sorrells M (2012). Identification of Ug99 stem rust resistance loci in winter wheat germplasm using genome-wide association analysis. Theor Appl Genet..

[42] Wang Y, Yu C, Cheng Y, Yao F, Long L, Wu Y (2021). Genome-wide association mapping reveals potential novel loci controlling stripe rust resistance in a Chinese wheat landrace diversity panel from the southern autumn-sown spring wheat zone. BMC Genomics..

[43] Yang X, Tan B, Liu H, Zhu W, Xu L, Wang Y (2020). Genetic Diversity and Population Structure of Asian and European Common Wheat Accessions Based on Genotyping-By-Sequencing. Front Genet..

[44] Tadesse W, Suleiman S, Tahir I, Sanchez-Garcia M, Jighly A, Hagras A (2019). Heat-tolerant QTLs associated with grain yield and its components in spring bread wheat under heat-stressed environments of Sudan and Egypt. Crop Sci..

[45] Sehgal D, Autrique E, Singh R, Ellis M, Singh S, Dreisigacker S (2017). Identification of genomic regions for grain yield and yield stability and their epistatic interactions. Sci Rep..

[46] Joukhadar R, Daetwyler HD, Gendall AR, Hayden MJ (2019). Artificial selection causes significant linkage disequilibrium among multiple unlinked genes in Australian wheat. Evol Appl..

[47] Zegeye H, Rasheed A, Makdis F, Badebo A, Ogbonnaya FC (2014). Genome-Wide Association Mapping for Seedling and Adult Plant Resistance to Stripe Rust in Synthetic Hexaploid Wheat. PLoS One..

[48] Aviles AC, Harrison SA, Arceneaux KJ, Brown-Guidera G, Mason RE, Baisakh N (2020). Identification of qtls for resistance to fusarium head blight using a doubled haploid population derived from southeastern united states soft red winter wheat varieties ags 2060 and ags 2035. Genes (Basel)..

[49] Rahman M, Davies P, Bansal U, Pasam R, Hayden M, Trethowan R. Marker-assisted recurrent selection improves the crown rot resistance of bread wheat. Mol Breed. 2020;40.

[50] Roelfs AP, Singh RP, Saari EE (1992). Concepts and methods of disease management.

[51] Bates D, Maechler M, Bolker B, Walker S, Christensen RHB, Singmann H, et al. Linear mixed-effects model using “Eigen” and S4, R Package Version 1.1–23. 2020. https://github.com/lme4/lme4/. Accessed 3 Oct 2020.

[52] Pritchard JK, Stephens M, Donnelly P. Inference of Population Structure Using Multilocus Genotype Data. 2000. http://www.stats.ox.ac.uk/pritch/home.html. Accessed 9 Sep 2020. 10.1093/genetics/155.2.945 PMC1461096 10835412

[53] Falush D, Stephens M, Pritchard JK (2007). Inference of population structure using multilocus genotype data: Dominant markers and null alleles. Mol Ecol Notes..

[54] Earl DA, vonHoldt BM (2012). STRUCTURE HARVESTER: A website and program for visualizing STRUCTURE output and implementing the Evanno method. Conserv Genet Resour..

[55] Evanno G, Regnaut S, Goudet J (2005). Detecting the number of clusters of individuals using the software STRUCTURE: A simulation study. Mol Ecol..

[56] Bradbury PJ, Zhang Z, Kroon DE, Casstevens TM, Ramdoss Y, Buckler ES (2007). TASSEL: Software for association mapping of complex traits in diverse samples. Bioinformatics..

[57] Liu K, Muse SV (2005). PowerMaker: An integrated analysis environment for genetic maker analysis. Bioinformatics..

[58] Letunic I, Bork P (2007). Interactive Tree Of Life (iTOL): An online tool for phylogenetic tree display and annotation. Bioinformatics..

[59] Allen GC, Flores-Vergara MA, Krasynanski S, Kumar S, Thompson WF (2006). A modified protocol for rapid DNA isolation from plant tissues using cetyltrimethylammonium bromide. Nat Protoc..

[60] Lipka AE, Tian F, Wang Q, Peiffer J, Li M, Bradbury PJ (2012). GAPIT: Genome association and prediction integrated tool. Bioinformatics..

[61] Breseghello F, Sorrells ME (2006). Association Mapping of Kernel Size and Milling Quality in Wheat (*Triticum aestivum* L.) Cultivars. Genetics..

[62] Sukumaran S, Reynolds MP, Sansaloni C (2018). Genome-wide association analyses identify QTL hotspots for yield and component traits in durum wheat grown under yield potential, drought, and heat stress environments. Front Plant Sci.

[63] Linear T, Mixed N, Models E, Fit D, Hmisc S. Package ‘ nlme .’ 2020.

